# Peroxide of Hydrogen

**Published:** 1888-08

**Authors:** 


ARTICLE VI.

### PEROXIDE OF HYDROGEN.

As peroxide of hydrogen is coming into such extensive use as a bleaching and remedial agent, the following, compiled from various sources, will be found of unusual interest. —[*Editor Dental Review.*

Mr. John G. Benedict, in a thesis submitted to the Massachusetts College of Pharmacy, gives a very comprehensive resume of the literation of hydrogen peroxide, in which it is stated that the substance was introdueed to common use in 1870 as a hair bleacher ; its value as a bleaching agent for animal substances being particularly great. As a germicide it also rates high. As a medicinal agent it was first proposed by Dr. B. W. Richardson in 1858. In May of the same year, Dr. W. B. Clark, of Indianapolis, published in the *Medical Era* his experience with it in a number of diseases, and Dr. W. A. Blackman, of Brooklyn, N. Y., read a similar paper before the New York Society for Medico-Scientific Investigation in July, 1866. Both of these authors spoke of its influence in destroying pus, and its curative influence in purulent ophthalmia, suppurative otitis media, gonorrhœa and leucorrhœa. Pean, a French surgeon, used it as a spray in ovariotomy. Dr. Day, of Australia, (*Lancet*, January 11, 1868), used an ethereal solution in a case of diabetes, which recovered. It is recommended in cases of septic dyspepsia and ulcerated teeth.

It is rated as sixty times more powerful as a bactericide than carbolic acid; twenty times as powerful as salicylic acid, and forty per cent. more potent than corrosive sublimate

Dr. J. Mount Bleyer draws attention to the efficacy of peroxide of hydrogen in a variety of diseases or conditions where a powerful oxidizing action is indicated. Thus, it is in many instances a most prompt antifermentative and antiseptic, exceeding in power even corrosive sublimate by many degrees. As a disinfectant it is superior to chlorine or sulphur. As an application in form of spray, to diphtheritic membranes, and other morbid conditions of the throat, it is of the highest benefit.

It may be either used in its undiluted, commercial condition, or in dilution.

Although hydrogen dioxide (peroxide of hydrogen) may be prepared from the ordinary commercial barium dioxide (binoxide of barium) yet the field is so unsatisfactory that it is under all circumstances advisable to employ a purified barium dioxide. This is prepared in the following manner:

Commercial barium dioxide is reduced to a very fine powder, and small portions of the latter are added, from time to time, to dilute hydrochloric acid (spec. grav. 1.050) until the latter is nearly neutralized. The solution having been cooled and filtered, it is treated with a small quantity of baryta water, whereby any ferric and manganic oxide, silica, or alumina are precipitated. As soon as a white precipitate of crystalline hydrated barium dioxide makes its appearance, the solution is filtered, and to the filtrate concentrated baryta water added; this causes a precipitation of crystalline, hydrated barium dioxide. It is well washed, and preserved, in its moist condition, in stoppered bottles.

To prepare hydrogen dioxide, small portions of the moist barium dioxide are gradually added to a cold mixture of one part of sulphuric acid and at least five parts of distilled water, taking care to prevent the temperature from rising beyond 20° C. (68° Fahr.), and suspending the addition when the liquid has only a slightly acid reaction. The precipitated sulphate of barium is allowed to deposit and the liquid filtered off. It is best to retain the slight excess of sulphuric acid since the compound keeps better. The solution thus obtained, which scarcely ever contains over five per cent. of hydrogen dioxide, may be concentrated either by exposing it in vacuo, over sulphuric acid, at a temperature of 15-20° C. (59-68° Fahr.), or it may be exposed to cold, and the frozen portion removed. The residuary liquid will be much more concentrated, as hydrogen dioxide does not freeze, even at -30° C. (-22° Fahr.). If the solution is allowed to evaporate over sulphuric acid, it may happen that bubbles of oxygen begin to be given off. In this case, the further addition of a few drops of sulphuric acid will arrest further decomposition.

Hydrogen dioxide is easily soluble in ether. If the aqueous solution be shaken with ether, the latter dissolves out the dioxide. This ethereal solution is much more stable than the aqueous solution, and may even be distilled without decomposition.

The quantity of hydrogen dioxide present in any aqueous solution may be determined by strongly acidulating the liquid with sulphuric acid, and adding from a burette a solution of potassium permanganate of known strength until the purple tint of the latter is no longer destroyed. In this case the following reaction takes place:

| $K_2Mn_2O_8$ — | $3H_2SO_4$ — | $H_2O_2$ = | $K_2SO_4$ — | $2MnSO_4$ — | $4H_2O$ — | $3O_2$ |
|---|---|---|---|---|---|---|
| Potassium permanganate. | Sulphuric acid | Hydro. dioxide. | Potassium sulphate. | Manganese sulphate | Water. | Oxygen. |

Each gram of potassium permanganate used corresponds to 0.108 grm. of hydrogen dioxide.

The reaction here quoted is given of the authority of Roscoe and Schorlemmer. According to others (Wislicenus, Schoene, Trommsdorff), the proportions are these.

$K_2MN_2O_8 — 3H_2SO_4 — 5H_2O_2 = K_2SO_2 — 2MNSO_4O — 8H_2O — 5O_2$

hence, one grm. of potassium permanganate would correspond to 0.541 grm. of hydrogen dioxide.

In commerce it is not sold in its pure condition, but in a dilute form. It is customary to designate the various grades of strengths by the volumes of gas which the particular solution is capable of yielding. Usually the commercial article is between "ten" and "twenty" volumes. By this it is understood that hydrogen dioxide, in its pure state, is a *liquid*, and not a gas; and the "ten volumes" is meant to say that the produce contains ten volumes of available oxygen; or, in other words, one measure of the commercial liquid can give off ten measures of oxygen. This corresponds to a strength of only three per cent. of hydrogen of dioxide. A "two volume" solution would be one which gives off twice its volume of oxygen, or which contains 0.6 per cent. of hydrogen dioxide.

The qualification, ten "volumes," originated some years ago, when the substance was first put upon the market and this qualification has been adopted in the price-lists of all countries.

It must be preserved in a cold, dark place, in glass-stoppered bottles, which should be small, if the consumption of the article is insignificant.

A new process for preparing peroxide of hydrogen has recently been patented in Germany by Siegfried Lustig of Breslau. It is briefly as follows:

Zinc amalgam is shaken with alcoholic solution of sulphuric acid and air. The mercury and the sulphate of zinc which is formed, and is precipitated in the alcoholic liquid, is removed by filtration, and the liquid concentrated in vacuo. It then represents an alcoholic solution of peroxide of hydrogen.—*Dental Advertiser*.

